# Is Higher-Order Misrepresentation Empirically Plausible? An Argument From Corruption

**DOI:** 10.3389/fpsyg.2022.804896

**Published:** 2022-03-16

**Authors:** Asger Kirkeby-Hinrup

**Affiliations:** ^1^Theoretical Philosophy, Department of Philosophy and Cognitive Science, Lund University, Lund, Sweden; ^2^Cognitive Neuroscience Research Unit, Center of Functionally Integrative Neuroscience, Aarhus University, Aarhus, Denmark

**Keywords:** higher-order theory, misrepresentation, consciousness, HOT theory, functionalism, materialism, higher-order misrepresentation

## Abstract

I present an empirically based argument for the plausibility of misrepresentation as posited by some higher-order theories of consciousness. The argument relies on the assumption that conscious states are generated by processes in the brain. The underlying idea is that if the brain generates conscious states then misrepresentation may occur. The reason for this is that brain states can be corrupted and, accordingly, a conscious state that is at least partly caused by a corrupted brain state may be a misrepresentation. Our body of knowledge from cognitive and behavioral neuroscience lends support to the idea that corruption of neural states is both possible and relatively frequent. If this is the case, I argue, it is plausible that occasionally such corruption may result in misrepresentation. I support this claim by arguing that the most prevalent theoretical alternative to the occurrence of misrepresentation—the so-called no-consciousness reply—seems less supported by our current knowledge in the domain of consciousness and cognition. This way of arguing for misrepresentation is different from other empirically based arguments in the debate because it is a meta-level argument resting on a general premise that most participants in the debate can accept.

## Introduction

One of the central questions for a theory of consciousness is what accounts for the difference between the mental states that are conscious, and those that are not. One group of theories argues that what provides an individual with a conscious experience of a mental state *p1* is the presence of another mental state *p2* that has *p1* as its intentional object. Because *p2* is about another mental state, *p2* is considered a higher-order state. Therefore, theories that explain consciousness in terms of higher-order states are called higher-order theories.^1^ Higher-order theories come in a wide variety of forms (Rosenthal, 1997; Carruthers, 2003; Lau, 2007; Gennaro, 2012; Coleman, 2015). One question that higher-order theories face is the distinctive roles played by *p1* and *p2*. For instance, is it possible that a higher-order state can misrepresent what mental state an individual is in? This is the question of misrepresentation. Those who endorse misrepresentation argue that the presence of *p2* is sufficient to generate a conscious experience of *p1* regardless of whether *p1* exists. Those who reject misrepresentation deny that a higher-order state—in itself—is sufficient for the individual to be in a conscious state.

In her seminal paper on misrepresentation, Neander (1998) distinguished between two types of misrepresentation. In *mild* misrepresentation, the higher-order state inaccurately represents the first-order state that it is about. Mild misrepresentation can be exemplified by a case where the individual has a first-order visual representation of *red* but the higher-order state makes the individual consciously experience *seeing blue*. In *radical* misrepresentation, the individual has a conscious experience of being in a state that she is not in. Radical misrepresentation occurs when a higher-order state exists but the lower-order state, that the higher-order state represents the individual as being in, does not. David Rosenthal, a proponent of misrepresentation, has criticized the distinction between mild and radical misrepresentation. Rosenthal argues that the line between mild and radical misrepresentation ultimately is arbitrary. Rosenthal (2004, p. 32) writes as:

“Suppose my higher-order awareness is of a state with the property P, but the target is not P, but rather Q. We could say that the higher-order awareness misrepresents the target, but we could equally well say that it’s an awareness of a state that does not occur. The more dramatic the misrepresentation, the greater the temptation to say the target is absent.”

So, Rosenthal argues that to ask whether a higher-order state is a mild or radical misrepresentation is misguided, because there is no non-arbitrary way to decide whether a higher-order thought (HOT) misrepresents its target or is about an absent target. How we describe the situation seems to be a matter of degree. If Rosenthal is right about this, it appears that the distinction between mild and radical misrepresentation collapses into radical misrepresentation.

Rosenthal holds that when misrepresentation occurs, the individual consciously experiences whatever the occurring higher-order thought represents her as experiencing. Rosenthal thinks that the possibility of discrepancies between a higher-order state and its target follows naturally from his theory, and is not only possible, but also fully coherent and theoretically harmless (Rosenthal, 2011, 2012). Many other proponents of HOT theory share this sentiment (e.g., Matey, 2006, 2011; Weisberg, 2010, 2011; Pereplyotchik, 2013; Berger, 2014).

In this paper, I present an empirically based argument for misrepresentation as posited by the higher-order theories of consciousness. This way of arguing for misrepresentation follows in the footsteps of earlier work in the debate by the advocates of misrepresentation and their opponents. For instance, Lau and Rosenthal (2011, p. 396) present empirical evidence from cognitive neuroscience, they argue provides the higher-order view with “substantial empirical plausibility.” What is implied in this line of thinking is that experimental and clinical findings carry evidential weight in the theoretical domain. Similarly, Lau and Brown (2019) take aim specifically at the issue of misrepresentation and present empirical cases they interpret as the occurrence of misrepresentation. They argue that the fact that empirical cases exist shows that misrepresentation is not just a hypothetical conceptual problem, but that a successful theory will need to explain these cases. They conclude that in this respect the higher-order theory fares better than its competitors. Not only proponents of higher-order theory have argued on empirical grounds in the debate. In fact, leveraging empirical evidence is becoming increasingly prevalent in debates between competing theories of consciousness. For instance, Kozuch (2014, p. 722) acknowledges that one virtue of the higher-order theories is the amenability to empirical confirmation or disconfirmation. Kozuch proceeds to argue that evidence from lesions to the prefrontal cortex tells against the higher-order account. Similarly, other participants in the debate (e.g., Beeckmans, 2007; Malach, 2011; Sebastián, 2014) have leveraged empirically based arguments against the higher-order theories. Finally, specifically in relation to misrepresentation, principled (but not currently feasible) ways of testing for this have been suggested (Kirkeby-Hinrup, 2020). However, some empirically based arguments proposed in support of misrepresentation recently also have had objections leveraged against them (e.g., Kirkeby-Hinrup, 2014, 2016; Brinck and Kirkeby-Hinrup, 2017).

The assumption that empirical data may arbitrate between philosophical theories that are on equal footing on conceptual grounds, i.e., by providing a basis for an inference to the best explanation is gaining traction within current debates on consciousness, even in light of warnings about the work empirical evidence can do for us in this regard (Hohwy, 2009; Fink, 2016; Klein et al., 2020; Overgaard and Kirkeby-Hinrup, 2021). Be that as it may, the assumption that empirical evidence has an important role to play is shared by many philosophers of mind, who disagree on almost everything else. For instance, Josh Weisberg (2013) suggests that the right way to approach the study of consciousness is through empirical data. Similarly, Brown (2012) suggests that any theory of consciousness that is going to be physically realistic must take into account the nature of the brain and its states. The importance of empirical evidence in the debate is underscored by Block (2007, p. 486), when he suggests that “the familiar default ‘method’ of inference to the best explanation, that is, the approach of looking for the framework that makes the most sense of all the data […]” is the best way to examine the relation between phenomenal consciousness and brain states. Recently, steps have been taken to attempt carrying this out in practice (Kirkeby-Hinrup and Fazekas, 2021). In later work (Block, 2009, p. 1120), further notes that “it is hard to avoid the impression that the biology of the brain is what matters to consciousness—at least the kind we have.”

In the rest of this paper, I will make the case that misrepresentation seems plausible given what we know about the brain. Unlike some previously proposed empirical arguments (e.g., Lau and Rosenthal, 2011; Lau and Brown, 2019), I do not take my starting point in neither a particular theory of consciousness, nor on a concrete empirical phenomenon. The argument I develop merely relies on the assumption that conscious states are generated in, and by, the brain. This assumption can be cashed out in different ways depending on how one conceives of the mind–brain relationship (see endnote iii). However, given that many theories of consciousness, and in particular most higher-order theories, are in the business of naturalizing the mind, they share this assumption. The underlying idea of the argument is that if the brain generates conscious states then misrepresentation may occur. The reason for this is that brain states can be corrupted and, accordingly, a conscious state that is at least partly caused by a corrupted brain state may be a misrepresentation. Call this the argument from corruption (AFC). The way the AFC argues for misrepresentation is different from other approaches to misrepresentation: the AFC is a meta-level argument resting on a general premise that most participants in the debate can accept (that conscious states somehow rely on brain activity). This means that the AFC does not take its starting point in a particular theory of consciousness but instead appeals to a view about the brain that is presumably shared by both proponents and opponents of misrepresentation. In support of this presumption, I gave examples above of a range of participants in the debate who appear to share this view.

## The Argument From Corruption

The AFC turns on a central assumption of those who are engaged in the project of naturalizing the mind. The assumption is that conscious states are generated in the brain, and consciousness thus depends on the integrity of its neural underpinnings. Given that this assumption is shared by most who oppose and who endorse misrepresentation, the AFC can proceed from common ground, thereby increasing the chance of making progress in the debate.

From this starting point, the AFC proceeds with the following question: If we think that conscious states are generated in the brain and we know that the physical makeup of the brain is susceptible to corruption, then why could corruption of the physical makeup of the brain not result in misrepresentation? Now obviously, as it stands, this question puts too much of the burden of proof on opponents of misrepresentation. So, the plausibility of the AFC will rely on an explication of the way corruption may result in misrepresentation as envisioned by the proponents of higher-order theory.^2^ Doing this will take a few steps, and as an initial move, it is useful to isolate the two premises that form the basis of the question.

1. Conscious states are generated in the brain.
2. Brain states are susceptible to corruption.

For the AFC to be plausible, it is necessary to justify each of these two premises. Regarding the first premise, this is usually taken for granted in the debates between competing (empirical) theories of consciousness. I will take this for granted here, but further support for this stance can be found in the introduction, as well as prominent publications (e.g., Doerig et al., 2020), and the whole debate about the localization of the neural correlates of consciousness (Lamme, 2003, 2004; Bor and Seth, 2012; Meuwese et al., 2013; Frässle et al., 2014; Kozuch, 2014; Boly et al., 2017; Odegaard et al., 2017; Michel and Morales, 2020).

In the next two sections, I will defend the second premise and provide two ways of conceiving of corruption at a general level (the philosophically inclined also may consult this^3^ lengthy endnote). With the two premises established, the central part of the question remains as: Why would it be impossible for corruption of the brain to result in misrepresentation? Readers familiar with the philosophy of science will surely recognize the induction problem lurking in the background here. Furthermore, asking opponents of a view to prove a negative is not a viable option. For these reasons, the crux of the AFC consists in pointing to the fact that corruption of the neural underpinnings of consciousness often results in a wide variety of surprising and counterintuitive phenomena. Given the prevalence and variety of such phenomena, the question then becomes whether we have any *empirical* reason to think misrepresentation could not result from corruption. The reason I accentuate *empirical* here is that some opponents of misrepresentation have theoretical or conceptual reasons for rejecting misrepresentation. Later (in The No-Consciousness Reply), I will consider, and reject, one prominent such reason.

### Are Brain States Corruptible?

In this section, I will briefly motivate the second premise of the AFC that brain states can be corrupted. Given that the topic of this text is the possibility of misrepresentation, the premise is in need of two major specifications. First, because misrepresentation requires that an individual is conscious of *something*, the kind of corruption that is relevant to the argument here cannot be such that it extinguishes consciousness. This means that cases of severe brain trauma that leave the individual unconscious, in a coma, or dead (although being clear cases of corruption of the physical makeup or function of the brain) cannot be invoked here. Second, the meaning of the term *corruption* must be clear. I here take corruption to be any kind of event in—or state of—the brain that results in abnormal processing, where this is defined in opposition to neurotypical subjects or processing. Findings in the fields of behavioral and cognitive neuroscience clearly support the possibility of corruption. Indeed, these fields are concerned with coupling observations of behavioral or cognitive performance with their neural underpinnings, and in many cases, behavioral and cognitive performance is abnormal (see, Gazzaniga et al., 2014 for an extensive review). Significant portions of the brain sciences take their starting points in examinations of various forms of abnormal cognitive or behavioral phenomena and investigate their neural causes. I take this fact to be sufficient to show that brain states are corruptible and sufficient to establish the second premise in general. To boot, many of the arguments leveraged in the debate between higher-order theories and the opponents rely on varieties of lesions or otherwise corrupted neural processing (e.g., the rare Charles Bonnet syndrome discussed in Lau and Rosenthal, 2011). I submit that this shows that the second premise is not controversial in the particular context of misrepresentation either. Nevertheless, it is conducive to understanding the AFC to get a more detailed view of the kind of corruption that may be relevant. Therefore, I will next present two types of corruption that appear relevant to the possibility of misrepresentation.

### Two Types of Corruption

To determine whether corruption can lead to misrepresentation we need to have a firmer grip on the notion of corruption and how it may work. Below, I distinguish between two types of corruption, based on considerations of how the brain processes and transfers information. The first type of corruption relates to the *transfer* of information across topographically distinct areas of the brain. I will call it *Corruption in Information Transfer* (CIT). CIT can be divided into two types.

The first type of CIT can be called *external* CIT. External CIT suggests that when information is transferred between distinct faculties something may go awry. What awry means in this context is that the information carrying signal is degraded or otherwise distorted in a way that affects the information embedded therein (e.g., as a result of degraded myelin sheaths or through the application of TMS). Even on the micro scale (such as in the signals from one neural ensemble to another), the transfer of information involves signals traveling across actual physical distances. It is also possible to envision external CIT occurring at the macro level. For instance, visual signals travel from the retina through the optic nerve to the visual cortex and beyond through the ventral and dorsal streams. To illustrate external CIT, imagine a messenger traveling with a bag of letters from one town to another. At one point, part of the road has been flooded and the bag of letters becomes wet, causing the ink of the letters to smudge. When the messenger arrives with the letters, their content has literally changed (how the recipients of the letters interpret the corrupted content is a separate question). In this analogy, the road is the neural pathway across which information is transferred. The two towns are the faculties between which information is transferred, and the letters are the information.

The second type of CIT may occur when information is transferred within a given faculty. When CIT is occurring in the transfer *within* a faculty, I will call it *internal* CIT. Because we know that many faculties (e.g., the visual system) are distributed across distinct topographical locations of the brain, information is often transferred internally within a faculty as processing is carried out. For instance, if one conceives of the visual system as comprising a faculty, it appears reasonable to say that this faculty is topographically distributed. It is distributed because visual input is processed in more than one place (e.g., the striate cortex and prestriate cortex). Furthermore, it is fairly well established that visual information is (initially) transferred sequentially through distinct topographical locations. At each stage in the sequence, the input received is processed for particular properties. Thus, the processing of, for example, spatial frequency and motion are handled separately. One might object that we should view each of these stages in the sequence as faculties on their own, rather than grouping them together into a large visual faculty. However, this is not an argument against CIT since on this view each of the faculties that belong to the visual system will still be distributed across several neural ensembles and thus will be susceptible to internal CIT when information is transferred among them.

For a useful analogy to illustrate internal CIT, imagine that a large corporation has hired a consultant to produce a report on some important issue. Once the report is received, it is passed through various departments of the company; each department adds their perspective and comments on the issue in question. The financial department adds some figures and some calculations of expenses and expected revenue. The marketing department produces an appendix concerning user segments, merchandize, advertising platforms, and so forth. Once the report has passed through all the relevant departments of the corporation it reaches the boardroom, the members of which will take some appropriate action based on the report. One can imagine that, at some point in the process of being shipped from one department to the next, a couple of pages of the report containing crucial notes or calculations get lost or become damaged. The upshot is that when the report finally reaches the boardroom its contents have been corrupted and the considerations of the board will be different than they would have been if the report had been intact. Importantly, the corruption of the report occurs *between* the departments, in the transfer of information. In the analogy, the report from the consultant is the information input to the faculty, the different departments are the internal parts of the faculty that process the input, and the boardroom is the output function of the faculty.

One might object that this analogy is too simple. Perhaps one finds it implausible that such an illustration maps into very complex neural circuitry. Perhaps one would insist that, for this analogy to be a reasonable description of neural processing, more than one department should be working on the report simultaneously. However, imagining a more complex corporation, with several input/output sections, and parallel processing, only increases the number of paths across which information must be transferred. This means that the possibility of corruption during transfer of information actually may increase with the complexity of the corporation (faculty). It worth noting that that parallel processing also may guard against corruption by maintaining the information in separate processing streams, which may decrease the impact of corruption to one stream. However, it is not clear that this will preempt the issues raised here, since it raises questions relating to how to arbitrate between diverging streams that originally contained the same information, possible corruption to such an arbitration mechanism, and retains the issue for cases where multiple processing streams do not obtain.

The second type of corruption one may envision is in the *processing* of the information of a given faculty or neural ensemble. Call this *Process Corruption* (PC). A range of cortical areas appears to be highly specialized. An example of specialized areas could be those comprising the visual system, where for instance V4 handles specific properties of the visual signal such as spatial frequency and orientation. When positing the possibility of PC, one envisions that the procedural integrity of faculties or neural ensembles may be corrupted. The result is that the faculties process information in abnormal ways. For a useful analogy to illustrate PC, we can imagine the corporation described above. As before, the report represents the information being transferred through different departments of the corporation. However, in PC, the corruption does not occur in the transfer from one department to another. To illustrate PC, we instead imagine that one of the departments makes a critical mistake. For instance, the financial department might use an erroneous model to predict the development of the market, or simply mistype numbers in the budget. Importantly, it is the processing by a particular entity that corrupts the information and yields the abnormal output.

One might wonder whether the possibility of PC pertains only to the structural level (e.g., faculties) or whether it can occur at lower levels as well (e.g., neural ensembles). Let us consider the structural level first. In visual agnosia, individuals fail to process some specific feature of visual input owing to corruption of the relevant specialized faculty in their visual system. The behavioral evidence and subjective reports from patients in cases of visual agnosia clearly indicate that the relevant feature is not processed normally. In many cases, the behavioral evidence and subjective reports are corroborated by neural imaging showing abnormalities in the relevant faculty. From this, it appears there is reason to think that PC can occur at least at the structural level.

Does it occur at lower levels as well? In response to this question, there are at least two lines of reply. The first line of reply asks whether, when PC occurs at the structural level, it is always an entire structure that is corrupted, or only some part of it. It does not seem that we need to posit that the entire structure must be corrupted for it to yield abnormal processing. Rather, it appears that corruption of some (perhaps integral) part of the structure may be sufficient for the structure to yield abnormal processing. If this is the case, then it appears that we have obtained low-level PC for free, simply by showing that structural PC is possible.

The second line of reply consists in switching the burden of proof to those who might want to argue that low-level PC cannot occur. Why, one may ask, should we not believe that PC could occur at low levels of processing? It seems there are reasons to think that it can (e.g., the first line of reply, and possibly others such as the delicateness of biological matter), but no obvious reasons to think that it cannot.

I do not purport that the two types of corruption considered here are the only types of corruption that can occur. Corruption might occur in ways not considered here. The purpose of the examples given here is merely to describe two fairly basic and uncontroversial types of corruption.

## The No-Consciousness Reply

If the AFC is convincing, this means that misrepresentation is empirically plausible. The operative word here is “empirically.” Conceptually, most agree that misrepresentation is possible, at least in so far as one endorses a representational theory of consciousness, given that a representational relation does not seem to entail the existence of what is represented (e.g., it is possible to represent the easter bunny). At the theoretical level however, several opponents of misrepresentation have denied that misrepresentation *in fact* obtains. Importantly, the motivations for this denial are theoretical rather than empirical. In this section, I will evaluate the so-called “no-consciousness reply” (Gennaro, 2004, 2006; Wilberg, 2010) given that this can be seen as reminiscent of an empirical claim. In brief, the no-consciousness reply accepts that occasionally a higher-order state may misrepresent its target first-order state but claim that in those cases no conscious event will follow, regardless of the cause of the misrepresentation. That is, if a higher-order state misrepresents its target state, the individual will not consciously experience being in the target state.

When applied to the AFC, the no-consciousness reply would amount to accepting the premises but rejecting the conclusion. In other words, proponents of the no-consciousness reply may accept that consciousness relies on processes in the brain (premise 1) and that brain processes are corruptible (premise 2) but reject that cases where misrepresentation occurs due to corruption can yield conscious states. One way to do this for proponents of the no-consciousness reply is to claim that precisely the neural processes underlying consciousness are *functionally fragile*, as it were. By “functionally fragile,” one would mean that *any* corruption of the neural processes that generate consciousness would result in *no* conscious states being generated. Thus, the claim is that exactly the processes underlying consciousness are, in fact, *not* corruptible, but only can be destroyed. In its theoretical formulation, the no-consciousness reply amounts to a stipulation, for instance through the positing of a necessary intrinsic relation (in Gennaro’s version between two proper parts of a complex mental state). Because this stipulation turns on an intuition that is not shared in the debate, its validity is problematic to assess and no consensus has emerged. Therefore, given that the claim is otherwise theoretically coherent and internally consistent, it appears the only way to evaluate objectively the functional fragility variant of the no-consciousness reply is to consider the empirical support for it.

There are neural processes and faculties that neuroscience suggests are empirically necessary for consciousness (Giacino et al., 2014). In addition to the necessary processes, there also are non-necessary processes involved in the production of consciousness at a given time. The non-necessary processes matter because in many cases these will modulate the *contents* of particular states of consciousness, even while they are not necessary for *being* conscious in the first place. Since misrepresentation is a matter of contents of states, what I here call non-necessary processes are highly relevant. For example, parts of the visual system may be damaged without neither consciousness, nor visual perception being extinguished completely, which goes to show that these processes cannot be *necessary* for consciousness and/or visual perception at the general level. Thus, non-necessary processes can be corrupted severely without extinguishing consciousness. This is supported by the fact that much of cognitive psychology and cognitive neuroscience is devoted exactly to investigating the symptoms of such corruption. An example of this is visual agnosia resulting from carbon monoxide poisoning (Gazzaniga et al., 2014, p. 225). The fact that certain processes involved in the generation of consciousness can be corrupted may suggest that some necessary processes might be corruptible as well. Inductive inference from the fact that many non-necessary processes are corruptible can be considered as support for this. Additionally, the fact that the necessary processes are instantiated in or identical to (see endnote *i* for this distinction) the same matter (*viz*. the brain) as the non-necessary processes lends some credibility to this inference.

Importantly, the AFC does not claim that corruption automatically generates misrepresentation. The claim here is not that any corruption automatically causes misrepresentation. If corruption is possible then it may destroy conscious states in some cases, just as is claimed by the proponents of the no-consciousness reply. In other cases, corruption may lead to degraded or otherwise flawed conscious states. Indeed, the list of the possible consequences of corruption may be very long. The AFC, I submit, is a reason to think that misrepresentation rightfully belongs on that list. The purpose here only is to make plausible that in some cases, corruption may result in misrepresentation. Given that there are at least inductive reasons based on the vast body of work in cognitive and behavioral neuroscience to think that corruption of both necessary and contingent neural processes may occur without extinguishing consciousness, the onus must be on the proponents of the no-consciousness reply to provide empirical support for their claim. Absent empirical reasons to think otherwise, claiming that exactly the processes underlying conscious states are functionally fragile appears *ad-hoc*.

## Concluding Remarks

I have put forward the AFC to argue that misrepresentation is empirically plausible. The AFC suggests that if corruption of the neural underpinnings of the generation of conscious states is possible, then occurrences of misrepresentation are plausible. Upon considering whether the no-consciousness reply could be leveraged as an objection to the AFC, I concluded that there appears to be no empirically based reason to endorse it. On the contrary, there is some inductive empirical support for the idea that the neural underpinnings of consciousness *can* be corrupted. If corruption is possible, this is reason to think that occurrences of misrepresentation in fact obtain.

It is worth mentioning that in my treatment of the no-consciousness reply, I mainly considered a version of the AFC suggesting misrepresentation may occur as a result of corruption of the necessary processes for generating consciousness. In addition to this, there is a weaker version of AFC positing that misrepresentation may occur as a result of corruption in non-necessary processes. The idea behind this weaker claim is that errors in early processing in the non-necessary processes (e.g., submodules of visual system) may propagate upstream and ultimately yield misrepresentation once the resulting states become conscious. While the weaker claim is certainly interesting, the purpose of the present text has been merely to suggest the empirical viability of AFC based on corruption of core processes involved in consciousness. However, there is no provision in the debates that the occurrence of misrepresentation must be the “fault” of the HOT or the faculty that generates HOTs. What matters for misrepresentation is that a HOT renders an individual conscious of being in a state the individual is not in? For mild misrepresentation, it matters whether there is an “original” first-order state that is misrepresented in some way, but this criterion can still be satisfied by AFC. In the introduction, I showed that most participants in the misrepresentation debate agree on the two basic premises that consciousness relies on the brain and that empirical evidence is pertinent to philosophical debates on consciousness. In concordance with these views, it seems the AFC has a role to play in our understanding of misrepresentation.

Importantly, what the AFC seeks to establish is only that misrepresentation is *plausible*. This is enough to put pressure on proponents of the no-consciousness reply or theories who otherwise object to misrepresentation. Some proponents of misrepresentation additionally may endorse the stronger claim that the frequency of misrepresentation is higher than the occasional malfunction. How exactly an argument for this further claim might look is not my concern here. Nevertheless, initially establishing the empirical *plausibility* of misrepresentation is an important step along the way to constructing such an argument.

## Data Availability Statement

The original contributions presented in the study are included in the article/supplementary material, and further inquiries can be directed to the corresponding author.

## Author Contributions

The author confirms being the sole contributor of this work and has approved it for publication.

## Funding

All funding for this paper under the post-doc award by the Swedish Research Council (Vetenskapsraadet) grant VR-2018-06595. Content of this paper was not influenced by the funding agency.

## Conflict of Interest

The author declares that the research was conducted in the absence of any commercial or financial relationships that could be construed as a potential conflict of interest.

## Publisher’s Note

All claims expressed in this article are solely those of the authors and do not necessarily represent those of their affiliated organizations, or those of the publisher, the editors and the reviewers. Any product that may be evaluated in this article, or claim that may be made by its manufacturer, is not guaranteed or endorsed by the publisher.

## Footnotes

^1^There are of course alternatives to the higher-order thought theories of consciousness. Each of these accounts are characterized by the endorsement of an alternative mechanism through which mental states are rendered conscious. Some of these accounts, in particular first-order (or reflexive) theories (Kriegel, 2003a,b, 2007; Lamme, 2004), are held to preclude the possibility of misrepresentation. Others, such as workspace (Baars, 1996, 1997, 2005; Mashour et al., 2020) and integration (Tononi, 2005; Tononi et al., 2016) theories, have not—to the best of my knowledge—been explicitly considered in relation the misrepresentation debate.

^2^One may, of course, have separate reasons for rejecting higher-order theory, and while the argument presented here—if successful—deals with one objecting to higher-order theory, it does not tell against competing theories of consciousness.

^3^For the philosophically inclined, here are two specified versions of the AFC deploying different standpoints on the way conscious states depend on brain activity. The positions most prevalent in current debates on consciousness are functionalism and materialism. Let us begin by framing AFC in functionalist terms (AFC-F).
*AFC-F*
P1) The neural matter of the brain is corruptible. P2) When neural matter is corrupted, any functions that are instantiated in it may malfunction. P3) Conscious states are functional states of the brain. C) Conscious states may malfunction. It appears that the AFC-F is valid as it is presented here. In the main text, I presented considerations in support of the first and second premises. The third premise follows from a functionalist view of the mind–brain relationship. Of course, the justification of the functionalist view may need independent motivation, but because we are taking it for granted here, P3 appears to be granted as well. Thus, it appears AFC is compatible with a functionalist view of the mind–brain relationship. Moving on to the version of AFC framed in materialist terms (AFC-M). Here is one way to do it:
*AFC-M*
P1) The neural matter of the brain is corruptible. P2) When neural matter is corrupted, any states of the matter may be corrupted. P3) Conscious states are (contingently) identical to brain states. C) Conscious states may be corrupted. Again, the argument appears valid. The first and second premises have been secured in above. However, one might object that on AFC-M the second premise appears too weak. The second premise appears too weak if one espouses a token-identity theory. Token identity entails that the relevant states under consideration in AFC-M are numerically identical to neural matter. If the mental states are numerically identical to neural matter, then it is not the case that the mental states *may* be corrupted. Rather, the objector will claim, any corruption of the neural matter entails corruption of states of the matter. I think this objection is valid. However, if the objection is correct, it only precipitates the strength of the AFC-M. For this reason, we can be satisfied with the weaker formulation of the second premise. The third premise merely reiterates the materialist standpoint. This might need independent motivation, but its validity is taken for granted here. The upshot of AFC-M is that it appears that corruption of the neural underpinnings of conscious states directly entails corruption of the mental states. Therefore, it appears as though the AFC has good footing if one adopts materialism. Finally, one might notice that the conclusions of AFC-F and AFC-M differ. The functionalist version concludes that conscious states *may malfunction*, while the materialist version concludes that conscious states *may be corrupted*. Therefore, one might ask whether both conclusions entail the possibility of misrepresentation. However, the difference in wording merely reflects the terminology of the framework, and the difference in wording therefore is inconsequential. The terms “malfunction” and “corruption” describe the same underlying phenomenon, *viz*. that something has gone awry in the neural machinery, which in turn may or may not affect the conscious experience of the individual.
